# A Neglected Animal Model for a Neglected Disease: Guinea Pigs and the Search for an Improved Animal Model for Human Brucellosis

**DOI:** 10.3389/fmicb.2018.02593

**Published:** 2018-10-31

**Authors:** Martha E. Hensel, Angela M. Arenas-Gamboa

**Affiliations:** Department of Veterinary Pathobiology, College of Veterinary Medicine and Biomedical Sciences, Texas A&M University, College Station, TX, United States

**Keywords:** *Brucella* spp., guinea pig (*Cavia porcellus*), animal model, comparative placentation, zoonotic disease

## Abstract

Brucellosis is a bacterial disease caused by species of the *Brucella* genus and affects a wide variety of domestic and wildlife species and is also an important zoonosis. The global burden of disease is difficult to assess but *Brucella* spp. have a worldwide distribution and are endemic in the Middle East, Africa, South America, and Asia. The clinical signs of fever and malaise are non-specific, and the available serological diagnostic tests lack a high degree specificity in endemic regions compared to other important public health diseases such as malaria. A better understanding of the pathogenesis of brucellosis through discoveries in animal models could lead to improved diagnostics and potentially a vaccine for human use. Mouse models have played an important role in elucidating the pathogenesis but do not replicate key features of the disease such as fever. Guinea pigs were instrumental in exploring the pathogenesis of brucellosis in the early nineteenth century and could offer an improvement on the mouse model as a model for human brucellosis.

## Introduction

Brucellosis is a disease caused by bacterial species of the *Brucella* genus, which are gram negative, facultative intracellular organisms (Corbel, 1997). Twelve species are currently recognized and infect a wide variety of domestic and wildlife species (Whatmore et al., 2016). Of the twelve species, *Brucella melitensis*, *B. abortus*, and *B. suis*. are considered important zoonotic agents and cause disease in both animals and humans (Corbel, 1997). Until recently, *Brucella* species were designated by the World Health Organization as neglected zoonotic agents, defined as disease entities that suffer from the trifecta of affecting a resource limited population, having a low political profile, and a correspondingly limited investment by governments and communities (Mableson et al., 2014). Diseases that are classified as neglected zoonoses are often endemic in regions that have multiple disease etiologies that have similar clinical presentations that can lead to misdiagnosis or under diagnosis of a particular disease entity (Mableson et al., 2014). Despite being removed from the list, brucellosis remains endemic in many parts of the world and is likely to remain a smoldering disease that causes morbidity in both animals and people.

*Brucellosis* in domestic animals is primarily recognized as a cause of reproductive failure with large numbers of bacteria shed in aborted fetuses, placentas, or in secretory products like milk (Corbel, 1997). Bacteria spill over from infected animals to people mainly through inhalation of bacteria created by handling aborted fetuses or placentas or through ingestion of unpasteurized milk or milk products (Corbel, 1997). While reproductive failure in domestic animals is a universally recognized disease manifestation, the incidence of reproductive disease in people is not well characterized. However, recent case studies and reviews suggest that the incidence of *Brucella*-induced reproductive disease in people is an under-reported phenomenon (Khan et al., 2001; Ali et al., 2016; Arenas-Gamboa et al., 2016).

The hallmark of acute clinical infection in people is relapsing fever accompanied by flu-like symptoms such as tiredness and depressed appetite (Corbel, 2006). Clinically, acute infection is secondary to colonization of the spleen and lymph nodes by bacteria that is manifested as splenomegaly and lymphadenomegaly (Young, 1983). Chronic infection can result in a “chronic fatigue syndrome” and/or osteoarticular disease such as inflammation of the spine (spondylitis), peripheral joints (arthritis), joint pouches (bursitis), bones (osteomyelitis) among others (Corbel, 2006). Because *Brucella* species are often transmitted directly from animals or their tissues to people, certain populations have a higher risk such as laboratorians, veterinarians, farmers, and abattoir workers (Corbel, 2006).

Better understanding of the pathogenesis of *Brucella* spp. is a strong step toward developing improved diagnostics and vaccine candidates for humans that can limit the impact of disease in endemic regions. Since *Brucella* was first isolated, animal models have served as important surrogates for understanding how *Brucella* species cause disease in humans. This review compares the most commonly used animal models and explores the potential of the guinea pig to serve as a model for brucellosis in people.

## Animal Models for Brucellosis

Several animal species have been used as surrogates for understanding aspects of *Brucella* pathogenesis in humans including mice, non-human primates (NHPs), rats, rabbits, and guinea pigs. Few studies have been conducted in rats and rabbits due to low disease susceptibility and transient nature of infection (García-Carrillo, 1990; Silva et al., 2011). The most commonly utilized small animal model is the mouse, which have been used to study the pathogenesis of infection as well as reproductive and osteoarticular disease (Bosseray, 1980, 1983; Tobias et al., 1993; Magnani et al., 2013). Mice are considered good models for chronic infection, and the course of infection has been extensively investigated (Grillo et al., 2012). I.p. inoculation is the most common route of inoculation, and mice develop a well characterized course of persistent infection that includes splenomegaly and peak replication in the spleen by 2–3 weeks post inoculation (Grillo et al., 2012). Other routes of inoculation, which are less frequently employed, include intravenous (i.v.), aerosol, oral, and intranasal (Bosseray, 1983; Enright et al., 1990; Kahl-McDonagh et al., 2007; Silva et al., 2011; Grillo et al., 2012; von Bargen et al., 2014). An advantage of the mouse model is the availability of reagents and genetic mutants that have made mice a valuable model for studying the pathogenesis of infection, but the mouse has several drawbacks (Grillo et al., 2012). While most studies use i.p. inoculation, this is an artificial means of inducing infection and is less biologically relevant than inhalational or oral routes of administration. The dose required to induce human infection has been historically estimated to be as low as 10–100 organisms, but the validity of this data is questionable (Pappas et al., 2006). Mice are considered naturally resistant to *Brucella* infection and need higher doses (>10^4^) when inoculated via aerosols or i.p. to demonstrate systemic disease (Kahl-McDonagh et al., 2007; Grillo et al., 2012). And finally, fever is a classic sign of human infection with *Brucella*, but mice do not develop fever at any dose or route of inoculation (Silva et al., 2011; Arenas-Gamboa et al., 2012). For example, B6.129s2-*Irf1^tm1Mak^*/J mice with implantable temperature transponders were challenged with 1 × 10^6^ CFU of *B. melitensis* biovar 1, *B. abortus* 2308, and *B. abortus* S19 and developed systemic infection but failed to develop fever (Arenas-Gamboa et al., 2012). Failure to develop fever in response to agents that induce fever in their natural hosts suggests that fever is not a physiologic response to infection in the mouse, which limits its use when investigating the human manifestation of disease.

In addition to mice, NHPs such as the rhesus macaque (*Macaca mulatta*) have been used as animal models for the study of brucellosis. In contrast to mice, NHPs share many aspects of the disease manifestation in humans including fever, reproductive failure, and colonization of the reticuloendothelial organs (Forest Huddleson and Hallman, 1929; Elberg et al., 1955; Percy et al., 1972; Mense et al., 2004; Yingst et al., 2010; Russell-Lodrigue et al., 2017). Macaques can be infected through routes that mimic natural transmission such as aerosols or ingestion of *Brucella*-laden milk. Following infection, bacteria can be recovered from the spleen, liver, lungs (aerosol inoculation), lymph nodes, and reproductive organs of males and females (Forest Huddleson and Hallman, 1929; Yingst et al., 2010; Russell-Lodrigue et al., 2017). Unfortunately, due to the high cost associated with animal husbandry and veterinary care, studies are often conducted with small numbers of animals, which limits the robustness of statistical analysis. Limited animal housing space in biosafety level three (BSL3) facilities, financial costs, and ethical considerations can make NHP studies less feasible for large scale studies. This model is also less practical in resource limited settings, which precludes research in a biologically relevant model in endemic countries.

## Guinea Pigs as Models for Brucellosis

Guinea pigs (*Cavia porcellus*) are caviomorph rodents that originated from the Andes of South America (Yarto-Jaramillo, 2011). Over the past 200 years, guinea pigs have proven to be a valuable animal model to study infectious diseases and are the model of choice for etiologic agents such as *Mycobacterium tuberculosis*, *Legionella pneumophila*, and Cytomegalovirus (Padilla-Carlin et al., 2008; McGregor and Choi, 2011). They have been chosen partially due to the similarity of immunologic components and reactions compared to humans such as complement and delayed type hypersensitivity reactions, respectively (Padilla-Carlin et al., 2008; McGregor and Choi, 2011). Most of the guinea pigs used in research are Hartley guinea pigs, which are an outbred strain of guinea pigs (García-Carrillo, 1990). Other outbred strains include Duncan-Hartley and English or American shorthair (García-Carrillo, 1990). The Strain 2 or 13 guinea pig are inbred strains, which have less genetic variation than the Hartley (García-Carrillo, 1990).

The guinea pig was the work-horse of the early *Brucella* studies because they could be infected by a variety of inoculation routes (subcutaneous, conjunctival, i.p., intranasal, i.v., vaginal, oral, or cutaneous scarification) and develop systemic disease (Meyer et al., 1922; Huddleson, 1943; Elberg and Henderson, 1948; Braude, 1951a; Druett et al., 1956; Phillips et al., 1956; Moulton and Meyer, 1958; Cuba-Caparo and Myers, 1973; García-Carrillo, 1990). From the 1910s to the 1960s, guinea pigs were considered the best laboratory animal for determining the virulence of different strains of *Brucella*, evaluating the efficacy of vaccine candidates, and investigating growth characteristics of *Brucella* (Fabyan, 1912; Huddleson, 1943; McCullough and Beal, 1951; McCamish and Elberg, 1962; García-Carrillo, 1990). The original studies inoculated guinea pigs via a subcutaneous or i.p. route with suspected infectious materials such as milk, placenta, or blood and evaluated the spleen and liver for colonization after 70-days (Fabyan, 1912; Meyer et al., 1922; Huddleson, 1943).

Guinea pigs are highly susceptible to infection with multiple species of *Brucella* including *B. suis*, *B. melitensis*, *B. abortus*, *B. neotomae*, and *B. ovis* (Cuba-Caparo and Myers, 1973; García-Carrillo, 1990). Infection has a negative impact on weight gain in the guinea pig, and they demonstrate other features of human brucellosis like fever and listlessness (Smillie, 1918; Meyer et al., 1922; Smith, 1926; Huddleson, 1943; Braude, 1951a; Braude and Spink, 1951; García-Carrillo, 1990). As an animal model, one of the intriguing aspects of the guinea pig is development of fever secondary to infection because this is not an aspect of infection that is replicated in the mouse model (Meyer et al., 1922).

**FIGURE 1:**
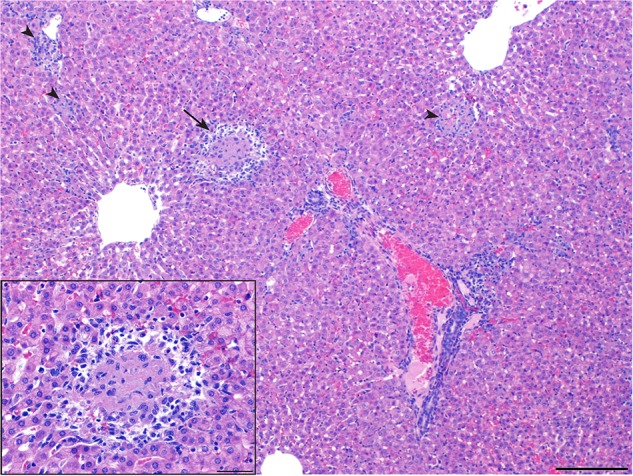
Liver of a guinea pig at 28 days of infection by *Brucella melitensis*. The guinea pig was infected intratracheally with 10^6^ CFU of *B. melitensis* 16M. Liver granuloma indicated by arrows (H&E, bar, 200 μm). Areas of histiocytic inflammation with necrotic hepatocytes are randomly distributed (arrowheads). Inset, higher magnification of area indicated by arrow demonstrating central necrosis surrounded by neutrophils and macrophages (H&E, bar, 50 μm).

Guinea pigs were heavily utilized in the first studies to describe the pathology and pathogenesis of infection and to compare the relative pathogenicity of the different strains (García-Carrillo, 1990). When guinea pigs were inoculated i.p. with 500,000 organisms from six strains of *B. melitensis*, *B. abortus*, and *B. suis*, infection caused splenomegaly, hepatic granulomas, and persistent infection up to 4 months post inoculation regardless of strain type (Braude, 1951a). While the lesion was similar despite the strain type, *B. suis* produced the most numerous and severe lesions (Braude, 1951a). A follow up study using a higher dose (6 × 10^8^) of *B. suis* i.p. identified lesions in the spleen that began as accumulations of polymorphonuclear cells (PMNs) with intracellular *Brucella* antigen as early as 24-h post inoculation and developed into foci of macrophage hyperplasia with occasional multinucleated giant cells by day seven. Splenic abscesses were commonly noted 100-days post inoculation and contained *Brucella* antigen within epithelioid macrophages (Moulton and Meyer, 1958). Spleen lesions in the guinea pig model are commonly identified, and the size of the spleen relative to body size is an useful indicator of infection when study guinea pigs are of similar size and sex (Garcia-Carrillo, 1977). The liver was affected in up to 60% of guinea pigs inoculated through an i.p. route (Braude, 1951a; Moulton and Meyer, 1958). The liver lesion in people is well-defined and is comparable to that which is reported in guinea pigs. From the aforementioned studies, the liver displayed a range of histologic lesions including granulomas, necrosis, abscesses, and periportal inflammation, which parallels descriptions of liver lesions in people (Figure 1; Braude, 1951a,b; Young et al., 2014).

Aerosols containing *Brucella* organisms are a common means of natural transmission (García-Carrillo, 1990; Corbel, 1997; Erdem et al., 2014). In order to investigate the potential of guinea pigs to serve as models for aerosol transmission, the first studies were attempted using the Henderson apparatus for generating aerosols (Henderson, 1952). This device delivered bacteria in a particulate cloud and was used to evaluate the respiratory pathogenicity of *B. suis* and *melitensis* in the guinea pig (Elberg and Henderson, 1948; Harper, 1955; Druett et al., 1956). Guinea pigs were exposed to 10^5^ CFU/ml via the Henderson apparatus and were euthanized after 30 days (Elberg and Henderson, 1948; Druett et al., 1956). Aerosol delivery of *B. suis* and *B. melitensis* resulted in systemic infection demonstrated by splenomegaly and recoverable CFU from the spleen, which demonstrates that guinea pigs are a useful model for evaluating the pathogenesis of aerosol inoculation. In the Elberg and Henderson study, bacteria were not recovered from the lungs nor were macroscopic or microscopic lesions seen in pulmonary tissue thus the respiratory tract is likely the portal of entry rather than a target of infection (Elberg and Henderson, 1948). Druett et al. (1956) collected samples for gross and histologic examination but did not publish those results, so the information is lost to history. Pulmonary disease in people is uncommonly reported even though aerosols are a common route of transmission (Young, 1995; Erdem et al., 2014). Body temperature was not evaluated in the guinea pig aerosol studies, so it is unknown whether this route of transmission would result in fever. A drawback to these early aerosol studies is that the dose of bacteria was calculated based on ventilation rate of the guinea pig and rate of delivery of the aerosol (Elberg and Henderson, 1948; Harper, 1955; Druett et al., 1956; Phillips et al., 1956). As such, it is impossible to know if the calculated dose was equivalent to the amount inhaled. Additionally, the Henderson apparatus was fastened around the head of the guinea pig to create a mini-aerosol chamber, which means the bacteria could have crossed the mucous membranes of the conjunctiva or been ingested. Despite these potential caveats, aerosol transmission was confirmed as a portal of entry for *Brucella* species that resulted in systemic disease in the guinea pig.

Due to the susceptibility of guinea pigs to multiple *Brucella* spp., they should be considered excellent translational models for testing vaccines or soluble antigens (Huddleson, 1942; Larson, 1949; Herzberg and Elberg, 1955; Keppie et al., 1972). It was expected that vaccines or antigens, which generated an active immune response in the guinea pig would be suitable for testing in humans and large animals after dose titration (Huddleson, 1942; Elberg et al., 1951; García-Carrillo, 1990). In particular, guinea pigs were instrumental for testing the safety and efficacy of the *Brucella melitensis* mutant Rev. 1 and *Brucella abortus* S19 vaccines for people (Herzberg and Elberg, 1955; McCamish and Elberg, 1962). Guinea pigs were also used to compare the protection provided by the various vaccine strains and antigens, such as S19, Rev. 1, 45/20 bacterins, etc., against field isolates and were used to determine if vaccination against one strain offered cross protection (Jones et al., 1958; Keppie et al., 1963; Alton, 1969; Cameron, 1979; Woodard et al., 1981; Hunter et al., 1989). These studies concluded that Rev. 1 was the most immunogenic of the vaccines tested, and immunization against one strain of *Brucella*, especially *B. suis*, was cross protective against challenge with *B. melitensis* or *B. abortus* (Keppie et al., 1963; Alton et al., 1967; Cameron, 1979). Further demonstrating the usefulness and value of the guinea pig, the World Animal Health Organization (OIE) lists guinea pigs as a suitable animal model to test master seed virulence, safety of S19 and Rev. 1, and toxicity of brucellin prior to their use in domesticated animals (Garin-Bastuji and Blasco, 2016).

After *Brucella* was initially described, researchers sought to characterize growth requirements, cell wall features, and virulence mechanisms of the bacterium (McCullough and Beal, 1951; Waring et al., 1953; Jones et al., 1965; Meyer, 1966, 1967). A typical growth experiment would evaluate the carbohydrate source, oxidation rate, carbon dioxide requirements, or iron requirement, etc., of a particular strain of *Brucella* and would inoculate the guinea pig with the test strains to determine if the bacteria retained virulence (McCullough and Beal, 1951; Waring et al., 1953; Jones et al., 1965). As an example, McCullough and Beal evaluated the ability of 12 strains of *Brucella* to utilize nine different carbon sources such as glucose, fructose, erythritol, etc. (McCullough and Beal, 1951). To further determine if the carbon source affected virulence, guinea pigs were inoculated with each of the test strains and assessed for colonization and gross lesions (McCullough and Beal, 1951). This seminal work identified erythritol as a preferred carbon source for *Brucella*. Follow up investigations into the role of i-erythritol on strain virulence in guinea pigs found that while erythritol is a preferred carbon source, it does not enhance virulence (Meyer, 1966, 1967).

## *Brucella* and the Reproductive Tract

Reproductive failure in cows was one of the first clues that led to the discovery of *Brucella abortus* (Huddleson, 1943). The incidence of reproductive disease in humans is a poorly investigated aspect of human brucellosis. However, as a neglected zoonosis, the incidence of brucellosis-related reproductive failure in women living in endemic regions is likely underdiagnosed. Case reports and retrospective analyses demonstrate that pregnant women who become infected and/or have serological evidence of infection with *Brucella* spp. are susceptible to adverse pregnancy outcomes, but the pathogenesis of disease is undefined (Young, 1983; Khan et al., 2001; Arenas-Gamboa et al., 2016). In order to better study the risks associated with *Brucella* and pregnancy in women, a biologically relevant model is paramount. One of the most important aspects to consider when choosing a model of reproductive disease is the type of placentation.

**FIGURE 2:**
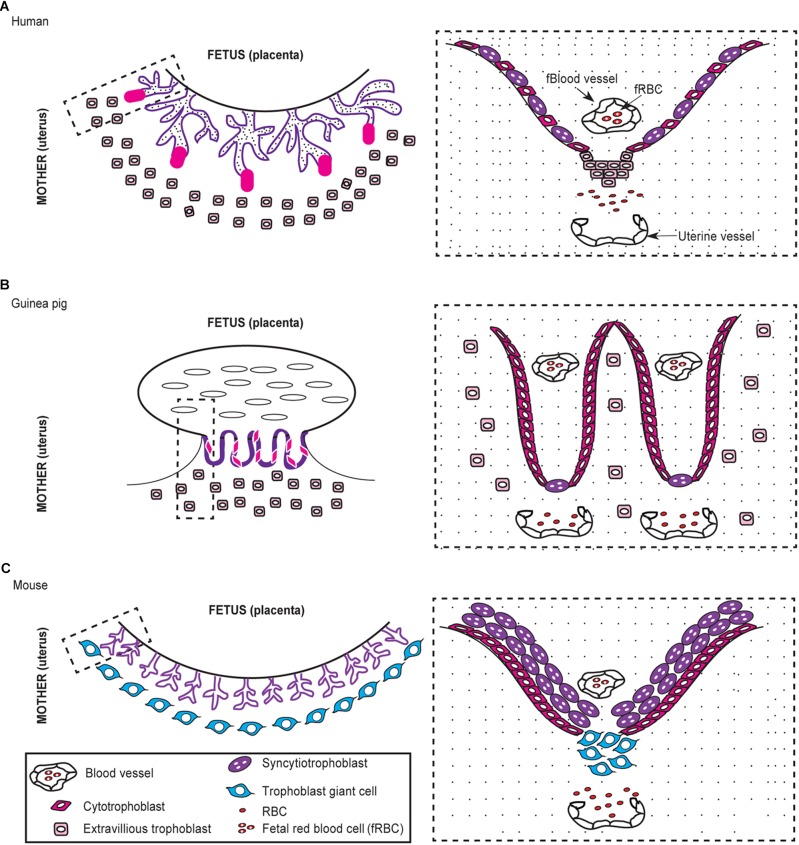
Comparative morphology of the human, guinea, and mouse placenta. The human and guinea pig placenta are hemomonochorial **(A,B)** while the mouse is hemotrichorial **(C)**. The dashed rectangle outlines the interface of the fetal-maternal blood supplies, which is shown in higher detail to the right.

The placenta is formed by a joining of the maternal endometrium with the trophectoderm from the embryo and is either classified by gross shape into four types (diffuse, multicotyledonary, zonary, or discoid/bidiscoid) or histological structure into three types (epitheliochorial, endotheliochorial, or hemochorial) (Furukawa et al., 2014). Humans, mice, and guinea pigs have a discoid hemochorial placentation (Carter, 2007; Mess et al., 2007; Furukawa et al., 2014). Hemochorial placentation is further subdivided into three subtypes based on the number of trophoblast layers that separate the fetal and maternal blood supplies (Furukawa et al., 2014). The mouse has a hemotrichorial placenta, which means three layers of trophoblasts separate the fetal and maternal circulation (Figure 2C; Furukawa et al., 2014). In contrast, humans and guinea pigs have a hemomonochorial placenta, and a single layer of trophoblasts separate the blood supplies (Figures 2A,B; Furukawa et al., 2014). Practically speaking, this means the interface between fetal and maternal blood streams is farther apart in mice. Along the same lines, trophoblast invasion is an important physiologic event that occurs during implantation and placentation (Silva and Serakides, 2016). Extensive trophoblast invasion implies that the trophoblasts almost completely replace the vascular endothelial cells of the uterine arteries, which allows for unimpeded blood exchange across the placenta to the fetus (Silva and Serakides, 2016). Mice have shallow invasion of trophoblasts, and so trophoblasts are not as involved in the remodeling of uterine arteries (Carter, 2007; Grillo et al., 2012).

The guinea pig placenta is one of the most physiologically similar to humans, but a few morphological differences exist such as a persistent yolk sac and subplacenta (Figure 2B; Kauffman and Davidoff, 1977). The yolk sac maintains pregnancy in the guinea pig and other rodents but is considered vestigial after the first trimester in humans (Kauffman and Davidoff, 1977). A unique feature of the guinea pig placenta is the subplacenta, which is located at the base of the individual fetal placental unit at the interface of the fetal and maternal blood supplies (Mess et al., 2007). The subplacenta is considered similar to the cell columns in the human placenta and is the site of trophoblast invasion (Mess et al., 2007). The guinea pig placenta has the advantage of being one of the most extensively investigated due its similarities to human placentation and has been used to investigate other reproductive tract pathogens such as syphilis (*Treponema pallidum*), chlamydia (*Chlamydia trachomatis*), gonorrhea (*Neisseria gonorrhoeae*), and more recently Zika virus (Padilla-Carlin et al., 2008; McGregor and Choi, 2011; Bierle et al., 2017). As such, the guinea pig could be a valuable model to investigate the underlying events that lead to pregnancy failure in women infected with *Brucella* spp. (Kauffman and Davidoff, 1977; Carter et al., 1998; Mess, 2007; Mess et al., 2007).

Another consideration in choosing a model of reproductive disease is the length of gestation and degree of fetal development in utero. Human gestation is approximately 280 days and is divided into trimesters. Murine gestation is approximately 19 days, and the offspring are born immature (altricial) with many developmental processes occurring postnatally (Carter, 2007). Gestation in the guinea pig is between 63 to 66 days, and guinea pigs give birth to mature (precocial) offspring that have undergone most of the developmental events in utero as occurs during human pregnancy (Kauffman and Davidoff, 1977). The longer length of gestation makes guinea pigs more suitable for pathogenesis studies of how infection affects both the placenta and the fetus.

## Small Animal Models of Reproductive Brucellosis

Pregnant mice have been used to evaluate the tropism and effects of *Brucella* on the gravid uterus (Bosseray, 1980, 1983; Tobias et al., 1993; Kim et al., 2005). Similar to the pathogenesis of cattle infected with *B. abortus*, the stage of gestation at which mice are infected with *Brucella* determines the outcome of infection (Bosseray, 1980). At days 7, 11, 13, and 15 of gestation the placenta was colonized by bacteria, but this did not result in abortions or fetal death (Bosseray, 1980). A second kinetics study discovered that mice inoculated i.p. at day nine with 10^5.7^ organisms developed severe necrosuppurative placentitis with evidence of fetal resorptions (Tobias et al., 1993). It is important to note that these two studies used different inoculum doses, which may account for differences in clinical results observed between the two similar studies. Mice that are inoculated at day 4.5 experience fetal death and resorption and thus the timing of inoculation is crucial to evaluating the response of the placenta to infection with *Brucella* in the mouse model (Kim et al., 2005). The mouse model has the advantage of short generation time, a large number of commercially available reagents, and transgenic mice (Carter, 2007). Furthermore, genes expressed during placental development are characterized in the mouse and are often analogous to those expressed during the development of the human placenta (Carter, 2007). However, the mouse does have key differences in placentation, degree of trophoblast invasion, and gestational length that may make it less suitable to exploring the pathogenesis of reproductive pathogen like *Brucella* (Carter, 2007).

The literature regarding the use of female guinea pigs as models for *Brucella* spp. is sparse and requires a review of the annals of the early 20th century. A study from 1918 found rare colonization of the ovary and uterus in response to i.p. inoculation of female guinea pigs with *B. abortus* from aborted bovine fetuses and placentas (Smillie, 1918). The field of study then languished until 1974 when Bosseray and Diaz used intramuscular injection to inoculate 14 pregnant guinea pigs with 5 × 10^4^ *B. abortus* 544 (Bosseray and Diaz, 1974). The guinea pigs displayed variable responses to inoculation with *B. abortus* including 5 abortions, one stillbirth with fetuses in less advanced stage of development, and 7 normal appearing live births (Bosseray and Diaz, 1974). This study demonstrated vertical transmission because nine offspring had recoverable CFU from the spleen and had seroconverted (Bosseray and Diaz, 1974). This is intriguing because it demonstrates that *Brucella* induces abortions/stillbirths in pregnant guinea pigs, and it mimics what has been documented in the case reports of *Brucella*-associated adverse pregnancy outcomes in women (Bosseray and Diaz, 1974; Young, 1983). The guinea pig could be a better model due to the placental homology and similarity in disease manifestation with the caveat that guinea pigs are larger, more expensive to house, and generally have fewer reagents available to investigate immunological events.

## Immunology of the Guinea Pig

Knowledge of the guinea pig immune response to infection has lagged behind the rapid advance of discovery made through the mouse model. Fewer reagents are commercially available for guinea pigs compared to mice, and individual labs are thus pressed to develop their own reagents and tests (McMurray et al., 2005; Padilla-Carlin et al., 2008; Schafer and Burger, 2012). Much of the information concerning the guinea pig response to infection has come from the field of tuberculosis research (Lyons et al., 2004; McMurray et al., 2005; Allen et al., 2008; Shang et al., 2011). The guinea pig genome has been sequenced and efforts are on-going to annotate it fully, which has increased the utility of the guinea pig as a model because it allows for the design of guinea pig specific reagents. Currently, guinea pig-specific transcriptome arrays can be developed using the genomic sequence available through the Ensemble database (Wali et al., 2014). An exhaustive list of the immune system of the guinea pig is beyond the scope of this review, but key features of the immune system that are relevant to the field of brucellosis are discussed.

Interleukin (IL)-8 is a chemokine that recruits neutrophils to sites of inflammation and is upregulated during *Brucella* stimulated inflammation *in vitro* (Zwerdling et al., 2009). Recombinant guinea pig IL-8 (rpgIL-8) was developed for a *Mycobacterium tuberculosis* model of neutrophil activation (Lyons et al., 2004). Neutrophils were stimulated with rpgIL-8 at physiologically relevant concentrations, which promoted chemotaxis and the elaboration of TNF-α (Lyons et al., 2004). Guinea pigs also express CXCR-1, the receptor for IL-8 (Takahashi et al., 2007). Mice do not express IL-8 or its receptor (CXCR-1), which is a disadvantage to this model for *Brucella* research (Padilla-Carlin et al., 2008).

Interferon-gamma (IFN-γ) and IL-12 are important activators of macrophages and are upregulated in response to *Brucella* infection (Baldwin and Parent, 2002). The role of IFN-γ in *Brucella* pathogenesis is an area of active interest. Two functions are proposed: (1) stimulates the production of the cytokine IL-12, which promotes classical macrophage activation and (2) reduces or prevents *Brucella* intracellular replication (Baldwin and Parent, 2002). The pattern of expression in human and guinea pig IFN-γ and IL-12 are very similar, and primer sets and clones are available to measure these cytokines in guinea pigs (Shiratori et al., 2001; Yamada et al., 2005).

Cell-mediated immunity is crucial to infection clearance and if the response is ineffective, it can lead to chronic infection (Skendros et al., 2011). The cell mediated response to *Brucella* involves a variety of cell types including CD4+ and CD8+ lymphocytes and antigen-presenting cells such as macrophages (Skendros et al., 2011). The guinea pig has the potential to be an excellent model for cell-mediated immunity due to homology in leukocyte antigens (major histocompatibility complexes [MHC]) and human group 1 CD1 proteins that other rodent models do not have (Dascher et al., 1999; Padilla-Carlin et al., 2008).

## Summary

The guinea pig has the potential to be an improved model for human infection with *Brucella* due to similarities in disease development, placental homology, and immunological responses to infection. One of the factors that has hampered the control of neglected zoonoses such as *Brucella* spp. is the availability of biologically relevant models that can improve understanding of the pathogenesis. Similar to *Brucella* spp., the guinea pig has been a neglected model that has suffered from a lack of investment. Individual labs have made strides toward developing the guinea pig as a model for infectious diseases, and the sequencing of the guinea pig genome offers an exciting potential for development of new reagents. This review highlights the historical role the guinea pig has played in exploring the pathogenesis of brucellosis, and it is the authors’ opinion that guinea pigs could offer an improved model for the investigation of human brucellosis, especially of the reproductive tract.

## Author Contributions

MH wrote the paper and developed the figures. AA-G conceived the paper and provided critical revision. All authors gave final approval for the manuscript.

## Conflict of Interest Statement

The authors declare that the research was conducted in the absence of any commercial or financial relationships that could be construed as a potential conflict of interest.
